# Clinical Management of Prostate Cancer in High-Risk Genetic Mutation Carriers

**DOI:** 10.3390/cancers14041004

**Published:** 2022-02-16

**Authors:** Roderick Clark, Jaime Herrera-Caceres, Miran Kenk, Neil Fleshner

**Affiliations:** 1 Division of Urology, University of Toronto, Toronto, ON M5G 1X6, Canada; miran.kenk@uhn.ca (M.K.); neil.fleshner@uhn.ca (N.F.); 2 Department of Urology, Penn State Health, Hershey, PA 17033, USA; jherreracaceres@pennstatehealth.psu.edu

**Keywords:** prostate cancer, germline genetic mutations, clinical management

## Abstract

**Simple Summary:**

Men with certain genetic differences are at much higher risks of developing metastatic and lethal prostate cancer. With the recent introduction of a new class of medications specifically targeted to these gene repair pathways (PARP inhibitors), it is critical to review the state of the literature surrounding the management of men with prostate cancer who have these genetic differences. We review the existing literature to address common clinical questions pertaining to this population. There is an urgent need for further research regarding clinical management in these scenarios as patients are increasingly seeking out genetic testing and consulting healthcare professionals for guidance.

**Abstract:**

Background: Prostate cancer is a leading cause of death. Approximately one in eight men who are diagnosed with prostate cancer will die of it. Since there is a large difference in mortality between low- and high-risk prostate cancers, it is critical to identify individuals who are at high-risk for disease progression and death. Germline genetic differences are increasingly recognized as contributing to risk of lethal prostate cancer. The objective of this paper is to review prostate cancer management options for men with high-risk germline mutations. Methods: We performed a review of the literature to identify articles regarding management of prostate cancer in individuals with high-risk germline genetic mutations. Results: We identified numerous publications regarding the management of prostate cancer among high-risk germline carriers, but the overall quality of the evidence is low. Conclusions: We performed a review of the literature and compiled clinical considerations for the management of individuals with high-risk germline mutations when they develop prostate cancer. The quality of the evidence is low, and there is an immediate need for further research and the development of consensus guidelines to guide clinical practice for these individuals.

## 1. Introduction

Prostate cancer is a significant cause of male cancer deaths [1]. Approximately one in eight men who are diagnosed with prostate cancer will die of it [2,3]. Since there is a large difference in mortality between low and high-risk prostate cancers, it is critical to identify individuals who are at high-risk for disease progression and death. It is well established that certain germline pathogenic variants confer an increased risk of both being diagnosed and dying of prostate cancer [4,5]. Contemporary data demonstrate that variants that disrupt the function of genes involved in DNA damage repair (e.g., *BRCA 1* and *BRCA 2*) are associated with aggressive prostate cancer [6,7,8]. The risk of metastatic disease is particularly striking among *BRCA 2* carriers. Furthermore, the identification of germline mutations in hereditary prostate cancer genes can help identify family members at high risk of cancer, providing the opportunity to pursue targeted genetic testing, tailored screening, and risk-reducing approaches along with the opportunity for personalized treatment recommendations.

While germline mutations are relatively rare, it is likely that they account for a significant proportion of population level risk burden beyond traditional factors (e.g., age and African heritage). Various testing panels assess many germline mutations, but we will consider the main elevation in prostate cancer risk to occur among individuals with germline mutations in (1) *BRCA 1*, (2) *BRCA 2*, (3) *ATM*, (4) *CHECK 2*, and (5) *HOX B13*. Estimates of the prevalence for each of these genes vary between 0.3 to 1.2% within the general population [9,10,11,12] but are much higher among individuals with prostate cancer [4]. Pritchard et al. identified that among men with metastatic prostate cancer, 11.8% had at least one presumed pathogenic germline mutation. Furthermore, they found that having a germline mutation was associated with a significantly increased risk of prostate cancer metastases (varying from a nonsignificant relative risk (RR) for *ATM* mutation (RR: 1.6 (95% CI: 0.8–2.8) to highly significant for individuals with *BRCA 2* mutations (RR: 26.7 (95% CI: 18.9–36.4)). Among those with germline mutations, 71% had a first-degree relative with prostate cancer. Clearly while these mutations are relatively rare in the general population, these individuals are at significantly increased risk to develop prostate cancer and disproportionately contribute to the burden of prostate cancer deaths.

The objective of our study is to review the important implications of high-risk germline mutations as they relate to (a) which prostate cancer patients to perform genetic testing on (b) prostate cancer prevention, (c) screening, (d) active surveillance for low-risk disease, (e) focal and minimally invasive treatments, (f) treatment of localized disease, (g) recurrent disease, and (h) treatment of metastatic prostate cancer.

## 2. Methods

We formulated the aforementioned clinical questions from our clinical experience running a high-risk prostate cancer clinic that directed literature reviews within each area. We allowed the state of the literature in each clinical area to dictate the types of studies that were included within each review.

To identify relevant articles for inclusion for each clinical question we performed an initial broad literature review to understand the current scope of evidence. Literature searches were conducted in MEDLINE, including Pre-MEDLINE EMBASE; BIOSIS Previews^®^; Web of Science^®^—with Conference Proceedings; and the Cochrane Central Register of Controlled Trials electronic bibliographic databases. All searches were restricted to studies published in the English language only. We did not perform a meta-analysis of any data from the identified research articles but focused on performing a narrative review. We hope that our review of the literature minimizes the risk of missing relevant articles within the literature. We did not restrict articles based on of year of publication.

Search queries were developed using combination of subject headings and free-text terms and tailored for each section. For all searches, editorials, news, and letters were excluded. The bibliographies of all relevant retrieved articles and reviews were also examined to identify further relevant articles.

## 3. Results

### 3.1. Clinical Question: Which Prostate Cancer Patient Should Be Tested for a Germline Genetic Mutation?

There continues to be significant variability in who is recommended to undergo germline genetic testing for prostate cancer risk (Table 1). The most widely cited recommendations come from the National Comprehensive Cancer Network, but recommendations are also regularly updated from the Philadelphia consensus conference [13,14,15]. The American Urological Association and European Association of Urology also offer similar suggestions for germline testing as those presented here and are recommended for review for practitioners caring for individuals with prostate cancer [16,17]. Again, there is some variation in the recommended genes for testing, but most recommend multi-gene panel testing using next generation sequencing technology to include *BRCA 1, BRCA 2, ATM, CHEK2, PALB2,* and mismatched repair genes (*MLH1, MSH2, MSH6,* and *PMS2*). The availability of government sponsored testing varies widely between jurisdictions but reductions in the price of next generation sequencing has resulted in the proliferation of commercially available testing options which typically cover the recommended genes with the addition of several others.

Clinical consideration: as per standing prostate cancer germline testing guidelines, all men who meet NCCN [14] guidelines should undergo germline genetic testing using an accepted laboratory method. The role of somatic testing for individuals with prostate cancer is an area of ongoing investigation. Several trials allow for the inclusion of individuals with somatic mutations; thus, future research will need to clarify the utility of this testing technique and if the risk of progression and death are similar for individuals with only these mutations as opposed to germline mutations.

### 3.2. Clinical Question: Are There Any Methods for the Prevention of Prostate Cancer among Individuals with an Identified High-Risk Germline Mutation?

There is no approved medical agent for the prevention of prostate cancer. Numerous randomized control trials were performed on potential agents including the 5-alpha reductase inhibitors (which act at the prostate gland to block the action of androgens in the prostate, e.g., Dutasteride and Finasteride), medications which manipulate the hormonal axis (e.g., Toremifene), nonsteroidal anti-inflammatory drugs (e.g., Refocoxib), and a number of nutritional supplements (e.g., Selenium, Vitamin E, and Soy). Results of these trials were mixed, with some being stopped because of cardiovascular toxicity (e.g., Refocixib [19]), some trials showing increased prostate cancer risk (e.g., Vitamin E [20]), and, most famously, two trials that showed a decreased risk of prostate cancer overall but an increased high grade disease in the treatment arm (PCPT and REDUCE trials [21,22]). This last association resulted in a US Federal Drug Agency black box warning for these medications [23]. There are several theories regarding the cause for this association [24], but these medications are not routinely used for prostate cancer prevention. Recently, there has been renewed interest in exploring the role of statins and metformin in the prevention of prostate cancer development, progression, and death [25,26], and while these agents have considerable promise for the general population, their specific effectiveness in individuals with high-risk germline mutations has not been evaluated.

Given the lack of evidence regarding the efficacy of these agents to prevent prostate cancer, these agents should not be recommended for prostate cancer prevention among individuals with high-risk germline mutations. Additionally, while there is no evidence among high-risk germline mutation carriers, 5-alpha reductase inhibitor use (e.g., for benign prostatic hyperplasia or male pattern hair loss) should be accompanied by a discussion of the risks and benefits of prostate cancer screening in this population due to the potential of increased risk of high-grade disease.

The idea of surgical prevention of cancer is well established in the breast and ovarian cancer literature among high-risk carriers [27]. There are some case reports of select high risk carriers who undergo prophylactic prostatectomy for disease prevention, but this should not be recommended outside of a clinical trial [28].

Clinical consideration: Currently, no agents are accepted for the prevention of prostate cancer among individuals at average or high risk. The use of 5-alpha reductase inhibitors among individuals with high-risk germline mutations should be accompanied by a discussion of the risks and benefits of these agents with a specific discussion targeted towards the risk of high-grade prostate cancer. Clinical trials of primary prevention are encouraged among these high-risk men.

### 3.3. Clinical Question: What Types of Prostate Cancer Screening Protocols Should Men with Identified High-Risk Germline Mutations Undergo?

Prostate cancer screening in the general population has been controversial. The discovery of the serum prostate specific antigen (PSA) in the early 1990s resulted in a sudden increase in population screening for prostate cancer with associated aggressive treatment that resulted in overtreatment among certain populations [29]. Three large-scale randomized control trials were performed with mixed results. The European ERSPC and Gotenberg studies found a 20–30% and 42% relative reduction in prostate cancer mortality [30,31], while the US PLCO trial showed no difference between the treatment and control arm (largely attributed to the presence of contamination of the control arm [32]). The results of these three trials resulted in the US preventative task force’s recommendation against PSA screening [33]. This has been subsequently updated to a recommendation for a discussion of the risks and benefits of screening in men aged between 55 and 69 and against screening for men over 70. It is important to recognize that these recommendations do not apply to men at increased risk for the development of prostate cancer.

Several organizations provide specific recommendations regarding screening for men at increased risk including the American Urological Association (AUA) and National Comprehensive Cancer Network (NCCN) and the American Cancer Society (ACS). The AUA recommended that men at increased risk discuss their individual cases with their doctors and states that their recommendations do not apply to men at increased risk. NCCN recommends that men with a germline mutation in *BRCA 1* and *BRCA 2* consider beginning shared decision making about PSA screening at the age of 40 and to consider annual screening [14]. ACS recommends starting a discussion about screening at the age of 40 for men at higher risk (e.g., those with more than one first degree relative who had prostate cancer at an early age) [34].

Fortunately, there is an ongoing clinical trial regarding the effectiveness of PSA screening among men with a *BRCA 1/2* mutation [35]. Interim results from three years of follow-up show that, compared to noncarriers, *BRCA 2* carriers had an increased incidence of prostate cancer, younger age of diagnosis, and more clinically significant tumors. The authors recommend that male *BRCA 2* carriers be offered systemic PSA screening. Alternative strategies for screening are also being explored, including the use of multi-parametric magnetic resonance imaging (MRI). Segal et al. [36] followed 188 *BRCA 1/2* carriers with PSA and MRI and found that MRI had the greatest benefit among younger carriers regardless of PSA level and that *BRCA* carriers aged older than 55 should use PSA screening and be referred for MRI if it is elevated.

We believe that prostate cancer screening should be tailored to patient risk tolerance after a discussion of risks and benefits. While uncertainty still exists, we believe that a baseline multiparametric MRI should be offered to high-risk carriers at the age of 40 along with a PSA level. Based on these results, the patient should be offered ongoing surveillance at regular intervals (including PSA assessment, digital rectal exam as well as periodic MRI assessment). The current prostate imaging-reporting and data system (PIRADS-2) classification does not take germline mutation status into account; thus, we would recommend increased suspicion towards MR-guided diagnostic biopsy for equivocal lesions. The role of MRI guided versus systemic biopsy is still being defined in the general population; thus, we cannot make a recommendation regarding differences between these modalities for high-risk carriers.

Clinical consideration: Optimal screening protocols for men with high-risk germline mutations have not been definitively established. As per NCCN guidelines, these men should consider earlier screening, including regular PSA and MR follow-up with a low threshold for prostate biopsy.

### 3.4. Clinical Question: Are Men with High-Risk Germline Mutations Candidates for Active Surveillance Treatment Protocols?

The historical overtreatment of men with low-risk disease has resulted in the widespread adoption of active surveillance strategies for men with low-risk localized prostate cancer. Numerous risk stratification systems exist for enrollment into active surveillance treatment, but all rely on a combination of factors from the PSA level, clinical stage, and biopsy results. Active surveillance treatment typically consists of a baseline biopsy followed but a confirmatory biopsy performed at 1 year and then subsequently around 5 years with regular PSA testing between biopsies. The role of MRI in active surveillance is still being defined but is likely to take a larger role in the future. Numerous centers have demonstrated that active surveillance is safe and acceptable for patients [37,38]. Between 36% and 73% of patients will transition from active surveillance to treatment over 10 years, but the development of metastatic disease remains low at 10 years (between 0.1 and 2.8%) [38].

There is a paucity of data on the safety or efficacy of active surveillance for men diagnosed with “low-risk” disease who have a high-risk germline mutation. A small cohort with limited follow-up is being evaluated in Israel. They are followed with PSA every 3 months and MRI at the time of 1-year confirmatory biopsy. At a median follow-up of 28 months, 67% of patients were free from disease progression or treatment [39]. In a larger series with longer follow-up, men with *BRCA 1/2* or *ATM* mutations were more likely to harbor aggressive prostate cancer [40]. It must be emphasized that *BRCA 1* and *2* mutations were aggregately assessed in this population; thus, it is unclear if *BRCA 2* subpopulations are particularly prone towards progression to metastatic disease and death from prostate cancer, as suggested by our own ongoing work.

Given the early state of research into the safety of active surveillance protocols for individuals with high-risk germline mutations, we feel that radical treatment should be the treatment of choice. For patients choosing active surveillance, risk categories should be made more stringent (e.g., no Gleason Grade Group 2 patients), potentially only including very low risk individuals [41]. Furthermore, we believe that if active surveillance is chosen, protocols should be augmented with the incorporation of multiparametric MRI and targeted biopsy to increase the identification of high-risk disease [42] as well as more regular evaluation after the confirmatory biopsy.

Clinical consideration: Men with high-risk germline mutations should not be eligible for active surveillance treatments using traditional selection criteria. The risk, benefits, and clinical uncertainty regarding this issue should be discussed with any man exploring active surveillance as a treatment modality for their prostate cancer.

### 3.5. Clinical Question: Are Men with High-Risk Germline Mutations Good Candidates for Either Focal or Whole Gland Minimally Invasive Treatments for Their Prostate Cancer?

Numerous alternatives to “traditional” treatments (e.g., surgery or radiotherapy/brachytherapy) for localized prostate cancer exist and include cryotherapy, high-intensity frequency ultrasound, and focal therapy options (e.g., partial prostate ablation with laser). These treatments are advantageous as they can be offered in patients who desire to avoid the side effect profile of traditional treatments. Currently, these treatments only have a conditional recommendation for the treatment of low or intermediate favorable prostate cancer as per the AUA/ASTRO/SUO risk stratification [41], with many being considered experimental in the standard patient population.

When considering minimally invasive treatments for prostate cancer, it is essential to differentiate between focal versus whole gland ablative therapies. A review of the broad range of potential treatment options is outside of the scope of this article, but we do not believe that individuals with high-risk germline mutations are candidates for focal treatments as the entirety of this prostate should be considered “at risk” for subsequent disease development and potential for metastatic spread. There is a need for more research in this area.

At this time, given that these treatments are experimental for individuals without germline mutations, whole gland ablative treatments should not be offered to individuals with a high-risk germline mutation outside the context of a clinical trial.

Clinical consideration: Focal or whole gland ablative therapies are considered experimental in men at average risk of prostate cancer and so should not be routinely offered to men with high-risk germline mutations outside the context of a clinical trial.

### 3.6. Clinical Question: What Is the Preferred Treatment for Clinically Localized Prostate Cancer among Men with High-Risk Germline Mutations?

Traditional treatments for localized prostate cancer broadly include surgery or radiotherapy. The efficacy and side effect profile has been well established for both surgery and radiotherapy [43]. Studies of the effectiveness of surgery or radiotherapy for individuals with high-risk germline mutations are all retrospective. Castro et al. [6] examined the tumor features and outcomes of 2019 patients with prostate cancer, which included 18 *BRCA 1* and 61 *BRCA 2* carriers. They found that *BRCA* mutation carriers were more likely to be diagnosed with high-risk disease (Gleason Grade group ≤ 4), advanced clinical stage disease (T3/4), involvement of local lymph nodes, or with metastatic disease at diagnosis. Five-year Cancer specific survival (CSS) and metastases-free survival (MFS) were significant improved in noncarriers compared to carriers (CSS: 96% vs. 82% MFS: 93% vs. 77%) [6]. In a subsequent publication of Castro et al. [44], they compared metastatic relapse and cause-specific survival among 67 *BRCA* carriers and 1302 noncarriers who received either radiotherapy or surgery. It is important to note that individuals who receive radiotherapy had more aggressive and locally advanced disease than those who were surgically treated (e.g., proportion of high-risk patients among carriers. Surgery: 34.4 vs. radiotherapy: 68.8%); thus, we should caution against comparing “apples to oranges”. When multivariable analysis was performed, treatment modality was not a significant predictor. It should be noted that when comparing CSS between carriers and noncarriers after surgical treatment, there was no significant difference between these groups at 10 years of follow-up, although the numbers appear clinically different (10-year CSS noncarriers: 95%; carriers: 79%). The difference between carriers and noncarriers was significant at 10-year follow-up (10-year CSS noncarriers: 81%; carriers: 47%). While it is difficult to make comparisons between treatment modalities this study does highlight that *BRCA* mutation carriers likely do significantly worse than noncarriers even with radical treatment. While the ideal treatment for localized prostate cancer has not been definitively established, we believe that these results speak to the fact that individuals who have high-risk germline mutations carriers should undergo treatment escalation for their disease.

Special discussion should be made for *ATM* mutation carriers and the risks of radiotherapy. Early work on the relationship between *ATM* mutations and prostate cancer found that there was a strong association between late complications of external beam radiotherapy and mutations of this gene [45,46]. Subsequent work has demonstrated that there is potential for increased therapeutic efficacy of radiotherapy, but, for known *ATM*, carriers care must be taken to minimize radiation dose to prevent toxicity or the potential for secondary malignancies [47]. The evidence around late toxicity and second malignancy is scant for the other germline mutations, but the best evidence in *BRCA* 1/2 carriers does not suggest any increased risk [48].

Clinical consideration: High-risk germline mutation carriers should be offered escalated treatment for their prostate cancer above what is typically recommended for noncarriers by clinical parameters (e.g., biopsy result, PSA). Further research is needed regarding the role of neo-adjuvant and adjuvant therapies within these populations.

### 3.7. Clinical Question: What Is the Preferred Treatment for Disease Recurrence (e.g., Biochemical Recurrence) Post-Definitive Prostate Cancer Treatments in Men with High-Risk Germline Mutations?

All definitions of disease recurrence post-surgery or radiotherapy rely on PSA definitions. After surgery, the most adopted definition is a PSA rise to 0.2 ng/mL or greater with a second confirmatory value [49]. After radiotherapy, the most accepted definition for recurrence is PSA nadir (baseline PSA level after stabilization post radiotherapy) plus 2 ng/mL [50]. Approximately 30–50% of patients will develop biochemical recurrence after surgery or radiotherapy [51,52,53]. While the natural history of progression to metastatic disease is dependent on multiple risk factors, many men have an indolent disease course. Commonly utilized treatments for biochemical recurrence include salvage radiotherapy with androgen deprivation after surgery and typically androgen deprivation therapy after radiotherapy.

As previously discussed, individuals with these germline high risk mutations are at increased risk to have poor prognostic disease at presentation, node positive disease, and to have metastatic disease [6]; thus, these individuals are at increased risk for biochemical recurrence after PSA nadirs or even to have PSAs remain detectable after surgical management. Given that these individuals have different responses to therapy than noncarriers, they may be candidates for early cisplatin-based chemotherapy, early use of Poly (ADP-ribose) polymerase (PARP) inhibitors, or early androgen deprivation therapy. While there is recent evidence that adjuvant radiotherapy is no better than early salvage radiotherapy among a non-selected population with adverse pathologic features post-surgical management [54], these results should be interpreted with caution in high-risk germline carriers who may benefit from earlier and more aggressive treatment. 

Clinical consideration: Men identified with high-risk germline mutations with recurrent prostate cancer (e.g., biochemical recurrence) should be treated by using an escalated approach compared to men at average risks of prostate cancer. There is a need for research into the role of early cisplatin-based chemotherapy or PARP inhibition in men who have biochemical recurrence after definitive treatment. 

### 3.8. Clinical Question: What Is the Optimal Treatment and Sequencing for Men with High-Risk Germline Mutations Who Develop Metastatic Prostate Cancer?

Approximately 5% of men present with (de novo) metastatic prostate cancer at diagnosis. Sixty-five percent % of men with biochemical recurrence after surgery will also develop metastatic prostate cancer in 10 years [55]. The current 5-year prostate-specific survival with metastatic prostate cancer is 29% [56]. The conventional treatment for metastatic prostate cancer is androgen deprivation therapy, which has resulted in the distinction between castrate sensitive (responds to androgen blockage) and castrate resistant (PSA risk or radiographic evidence of progression of disease) metastatic prostate cancer. Historically, 10–20% of patients with metastatic prostate cancer develop castrate resistance within 5-years [57] at a median time between 13 and 19 months [58].

For high-risk germline carriers, it is known that they are at risk of progressing from castrate sensitive to resistant metastatic disease earlier than noncarriers [59,60,61]. Once carriers progress to castrate resistance, there are mixed data about how they perform compared to noncarriers. Several retrospective studies showed that patients with castrate resistance either have worse overall survival [59], have better progression free survival [62], or that there is no difference compared to non-carriers [4]. This could be a consequence of differences in disease burden or their treatment with either cisplatin-based chemotherapy or PARP inhibitors. PROREPAIR-B [60] is an ongoing prospective study for evaluating the outcomes of patients with metastatic castrate resistant prostate cancer. They have demonstrated that mutations in *BCRA 2* have worse outcomes, but the association is not clear in other germline mutations.

There has been a surge in interest in the utility of both cisplatin-based chemotherapy and PARP inhibitors and their roles in metastatic prostate cancer. This is beyond the scope of this article, but we would direct readers to the excellent review of this topic presented by Lozano et al. (BJC 2020), which provides a review and highlights ongoing trials. We highlight several important trials within this space that are ongoing (Table 2).

Clinical consideration: All men who present de novo or develop metastatic prostate cancer should undergo germline genetic testing. Individuals with a high-risk germline mutation should consider enrolling in a clinical trial to establish the optimal sequencing of agents in this population. Several clinical trials exploring the early or combination PARP inhibitors among these individuals are ongoing.

## 4. Conclusions

Our recommendations for clinical considerations based on the low-level of evidence are summarized in Table 3. The identification and paradigm for managing patients with genetic mutations and prostate cancer prevention and therapy will evolve in the coming decade. Aside from the role of PARP inhibition in CRPC, novel data are required to provide level 1 guidance. Herein, we provide pragmatic considerations for clinical scenarios of interest. Clinical practice is rapidly entering the era of personalized medicine; thus, we must accelerate research efforts to effectively integrate clinical genetics into urologic oncology practice.

## Figures and Tables

**Table 1:** Varying guidelines for genetic testing in prostate cancer (as presented from Clark et al. 2021 [18]).

Category	NCCN HBOPC Version 1.2021	NCCN Prostate Version 2.2020	Philadelphia Consensus Conference	American Urological Association	European Association of Urology
Metastatic disease	Metastatic PrCA	Metastatic PrCA	Metastatic PrCA (castrate resistant or sensitive; Recommend)	Metastatic PrCa (castrate resistant or sensitive)	Consider in metastatic PrCa
Histology	Intraductal/cribriform histology	Intraductal/cribriform histology	Intraductal/ductal pathology (Consider)		
Grade, Stage, PSA	High risk, very high risk group - ≥Stage T3a - ≥Grade Group 4 - PSA > 20 ng/mL	High risk, very high risk, or regional	Advanced disease (T3a or higher; Consider) Grade Group 4 (Gleason sum 8) or above (Consider)	High risk localized and a strong family history of other specific cancers	High risk PrCa who have a family member diagnosed with PrCA at age <60 years
Ancestry	Ashkenazi Jewish ancestry	Ashkenazi Jewish ancestry	Ashkenazi Jewish ancestry (Consider)		
Family History	Personal Hx PrCA with: (a) ≥1 close relative with breast <50 y and/or ovarian and/or pancreatic and/or metastatic/intraductal/cribriform PrCA at any age (b) ≥2 close relatives with breast or PrCA (any grade) at any age	Positive family history of cancer: (a) Brother or father or multiple family members diagnosed with PCA (not clinically localized Grade Group 1) at <60 y of age or who died from PrCA, *OR* (b) ≥3 cancers on the same side of the family, especially diagnosed ≤50 y: bile duct, breast, CRC, endometrial, gastric, kidney, melanoma, ovarian, pancreatic, PrCA (not clinically localized Grade Group 1), small bowel, or urothelial cancer	One brother/father or ≥2 male relatives: (a) Diagnosed with PrCA at age <60 y (Recommend) (b) Any of whom died of PrCA (Recommend) (c) Any of whom had metastatic PrCA (Recommend) FH of other cancers: ≥2 cancers in HBOC or Lynch spectrum in any relatives on the same side of the family (especially if diagnosed at <50 y; Consider)	risk localized and a strong family history of other specific cancers	Men with a family history of high-risk germline mutations or a family history of multiple cancer on the same side of the family

**Table 2:** Ongoing clinical trials for metastatic prostate cancer that may benefit men with high-risk germline mutations.

Trial Name	Inclusion Criteria	Intervention	Outcome
A Study of Niraparib in Combination with Abiraterone Acetate and Prednisone Versus Abiraterone Acetate and Prednisone for Treatment of Participants with Metastatic Prostate Cancer (MAGNITUDE)	Participants with metastatic castration-resistant prostate cancer and homologous recombination repair gene alteration (also includes a cohort without a mutation)	Combination of niraparib or matching placebo and abiraterone acetate plus prednisone	Effectiveness of niraparib in combination with abiraterone acetate plus prednisone compared to AAP and placebo
A Study of Niraparib in Combination with Abiraterone Acetate and Prednisone Versus Abiraterone Acetate and Prednisone for the Treatment of Participants with Deleterious Germline or Somatic Homologous Recombination Repair (HRR) Gene-Mutated Metastatic Castration-Sensitive Prostate Cancer (mCSPC) (AMPLITUDE)	Patients must have appropriate deleterious homologous recombination repair gene alteration and metastatic castrate sensitive prostate cancer	Participants will receive niraparib, abiraterone acetate plus prednisone versus matching placebo with abiraterone acetate plus prednisone	To determine the effectiveness of combination of niraparib with abiraterone acetate plus prednisone compared with abiraterone acetate plus prednisone
A Study of Rucaparib in Patients with Metastatic Castration-resistant Prostate Cancer and Homologous Recombination Gene Deficiency (TRITON2)	Patients must have a deleterious mutation in *BRCA1/2* or *ATM*, or molecular evidence of other homologous recombination deficiency with metastatic castrate resistant prostate cancer	Oral rucaparib (monotherapy)	how patients with metastatic castration-resistant prostate cancer, and evidence of a homologous recombination gene deficiency, respond to treatment with rucaparib
Study of Olaparib (Lynparza™) Versus Enzalutamide or Abiraterone Acetate in Men with Metastatic Castration-Resistant Prostate Cancer (PROfound Study)	Patients must have a qualifying homologous recombination deficiency mutation in tumor tissue and metastatic castrate resistant prostate cancer	Subjects will be administered study treatment orally versus enzalutamide OR abiraterone acetate	efficacy and safety of olaparib versus enzalutamide or abiraterone acetate in subjects

**Table 3:** Summary of clinical considerations.

Clinical Question	Clinical Consideration	Level of Evidence/Justification
Which prostate cancer patient should be tested for a germline genetic mutation?	As per standing prostate cancer germline testing guidelines, all men who meet NCCN guidelines should undergo germline genetic testing using an accepted laboratory method (Table 1).	Clinical guidelines on appropriate populations for testing are well established and consistent across guidelines from several organizations.
Are there any methods for the prevention of prostate cancer among individuals with an identified high-risk germline mutation?	Currently, no agents are accepted for the prevention of prostate cancer among individuals at average or high risk.	Extensive research has been performed on medication prevention of prostate cancer but has not been performed in high-risk genetic populations.
What types of prostate cancer screening protocols should men with identified high-risk germline mutations undergo?	These men should consider earlier screening including regular PSA and MR follow-up with a low threshold for prostate biopsy.	Level 1 evidence is accumulating regarding this question and indicates that more intensive screening among these individuals is justified.
Are men with high-risk germline mutations candidates for active surveillance treatment protocols?	Men with high-risk germline mutations should not be eligible for active surveillance treatments using traditional selection criteria.	There is very little research in this area and, thus, active surveillance should be considered only in clinical trials for these populations.
Are men with high-risk germline mutations good candidates for either focal or whole gland minimally invasive treatments for their prostate cancer?	Focal or whole gland ablative therapies are considered experimental and so should not be routinely offered to men with high-risk germline mutations outside the context of a clinical trial.	Should be considered only in clinical trials for these populations.
What is the preferred treatment for clinically localized prostate cancer among men with high-risk germline mutations?	High-risk germline mutation carriers should be offered escalated treatment for their prostate cancer above what is typically recommended for noncarriers by clinical parameters (e.g., biopsy result, PSA).	Only retrospective evidence exists regarding this issue and thus these men should be considered to be at high-risk for disease recurrence and progression.
What is the preferred treatment for disease recurrence (e.g., biochemical recurrence) post-definitive prostate cancer treatments in men with high-risk germline mutations?	Men identified with a high-risk germline mutations with recurrent prostate cancer should be treated using an escalated approach compared to men at average risk of prostate cancer.	Only retrospective evidence exists regarding this issue and, thus, these men should be considered to be at high-risk for disease progression and death from prostate cancer.
What is the optimal treatment and sequencing for men with high-risk germline mutations who develop metastatic prostate cancer?	Individuals with a high-risk germline mutation should consider enrolling in a clinical trial to establish the optimal sequencing of agents in this population.	Level 1 evidence is accumulating for the use of these agents in high-risk populations but ideal sequencing is still under investigation.
